# The complete mitochondrial genome of North Island brown kiwi (*Apteryx mantelli*)

**DOI:** 10.1080/23802359.2016.1186511

**Published:** 2016-12-26

**Authors:** Jia Liu, Qing-xia Ding, Li-zhi Gao

**Affiliations:** a Faculty of Life Science and Technology, Kunming University of Science and Technology, Kunming, China;; b Plant Germplasm and Genomics Center, Germplasm Bank of Wild Species in Southwest China, Kunming Institute of Botany, Chinese Academy of Science, Kunming, China

**Keywords:** *Apteryx mantelli*, mitochondrial genome, evolutionary relationships

## Abstract

Here, we report the complete mitochondrial genome sequence of North Island brown kiwi(*Apteryx mantelli*). The genome is found to be 16,694 bp in length and has a base composition of A (30.74%), G (13.46%), C (26.50%), and T (29.30%). Similar to other *Apteryx* species, it contains a typically conserved structure including 13 protein-coding genes, 2 rRNA genes, 1 control region (D-loop), and 22tRNA genes. The proportion of coding sequences with a total length of 11,431 bp is 68.47%, which encodes 3776 amino acids. All protein-coding genes started with Met, and *ND2*, *COX2*, and *COX3* ended by TAA as a stop codon. The lengths of 12S ribosomal RNA and 16S ribosomal RNA are 973 bp and 1596 bp, respectively. The length of control region is 1112 bp, ranging from 15,583 bp to 16,694 bp. The complete mitochondrial genome sequence provided here would be useful for further understanding the evolution of ratite and conservation genetics of *A. mantelli*.

Kiwi, belonging to very old bird group, is not only the national bird of New Zealand, but also the smallest bird among the flightless ratites (Prinzinger & Dietz 2004). The genus *Apteryx* consists of three kiwi species and five subspecies, of which the North Island brown kiwi (*Apteryx mantelli*) is the most common one. The populations of *A. mantelli* is decreasing 3% every year (Holzapfel et al. 2008), and thus, International Union for Conservation of Nature (IUCN) has included it in the Red List of Endangered species (Version 3.1, 2012). Although two complete mitochondrial (mt) genomes belonging to *Apteryx* have been determined (Haddrath & Baker 2001), the *A. mantelli* mt genome sequence hasn’t been reported.

Here, we sequenced and characterized the complete mt genome of *A. mantelli*. The mt genome reads were filtered from high through-output genome sequencing project of *A. mantelli* (Duc et al. 2015) from European Nucleotide Archive (Accession: PRJEB6383) by Blast (Altschul et al. 1997) using the *A. haastii* (NC_002782) mt genome sequence (Haddrath & Baker 2001) as a reference. The data were from three sequenced *A. mantelli* female individuals, which originate from the far North (kiwi code 73) and central part – Lake Waikaremoana (kiwi code AT5 and kiwi code 16–12) of North Island, New Zealand (Duc et al. 2015). About 19 Mb mt reads were obtained and assembled by using the CLC Genomic Workbench (version 3.6). Annotation was performed with DOGMA (http://dogma.ccbb.utexas.edu). rRNA, protein-coding, and tRNA genes were predicted by using default parameters (Wyman et al. 2004). The mt genome sequence of *A. mantelli* with the annotated genes was deposited in GenBank under the accession number of KU695537. A total of 13 complete mt genomes were sampled for phylogenetic analysis (Härlid et al. 1998; Haddrath & Baker 2001; Cooper et al. 2001; Pratt et al. 2009). MAFFT (version 7.158) was used for whole-genome alignments. ML analysis was performed using ‘fast bootstrap’ algorithm under GTRGAMMAX model replicated 100 times, which was implemented in RAxML (version 7.2.6).

Whole mitochondrial genome sequence of *A. mantelli* has a circular genome of 16,694 bp, containing 13 protein-coding genes, 2 rRNA genes, 1 control region, and 22tRNA genes. The contents of A, G, T, and C are 30.74%, 13.46%, 26.50%, and 29.30%, respectively. AT and GC contents of mt genome are 57.24% and 42.76%, respectively. The proportion of coding sequences with a total length of 11,431 bp is 68.47%, which encodes 3776 amino acids. All protein-coding genes started with Met. Remarkably, *ND2*, *COX2*, and *COX3* ended by TAA as stop codon. The lengths of 12S ribosomal RNA and 16S ribosomal RNA are 973 bp and 1596 bp, respectively. The length of D-loop is 1112 bp, ranging from 15,583 to 16,694 bp.

Phylogenetic analysis included mt genome of *A. mantelli* and the other 10 species that are from the order Struthioniformes, Dinornithiformes, Rheiformes, Casuariiformes, and Apterygiformes, which belong to Ratitae, Using *Trogon viridi* (Trogoniformes) of Trogonidae family as an outgroup. Maximum-likelihood (ML) analysis exhibited that *A. mantelli* clustered with the other two *Apteryx* species, *A. haastii* and *A. owenii*, highly supported by a bootstrap value of 100 (Figure 1). The evolutionary relationships of these analyzed species are consistent with previously reported results (Mitchell et al. 2014). The newly determined mt genome will help to understand the evolution of ratite.

**Figure 1.**
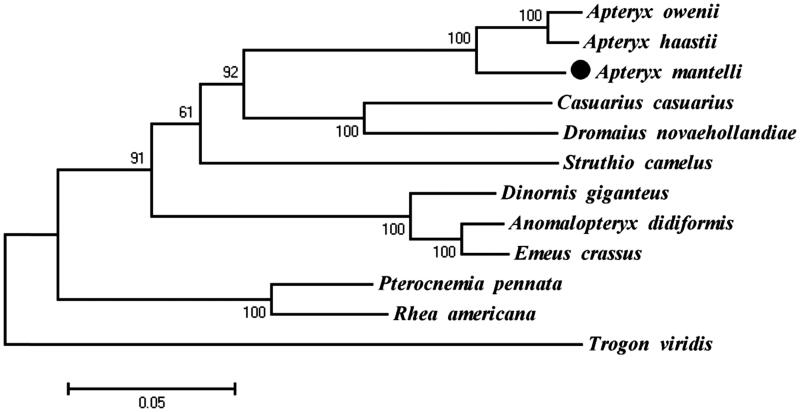
Maximum-likelihood (ML) phylogenetic tree of *A. mantelli* and the other 11 species using *T. viridis* as an outgroup. Number above each node indicates the ML bootstrap support values. All 12 species’s accession numbers are listed as below: *A. mantelli* KU695537, *A. haastii*NC_002782, *A. owenii*NC_013806, *Casuarius casuarius*NC_002778, *Dromaius novaehollan*NC_002784, *Struthio camelus*NC_002785, *Dinornis giganteus*NC_002672, *Anomalopterux didiformis*NC_002779, *Emeus crassus*NC_002673, *Pterocnemia pennata*NC_002783, *Rhea Americana*NC_000846, *T. viridis*NC_011714.
